# Revisiting Local Descriptors via Frequent Pattern Mining for Fine-Grained Image Retrieval

**DOI:** 10.3390/e24020156

**Published:** 2022-01-20

**Authors:** Min Zheng, Yangliao Geng, Qingyong Li

**Affiliations:** Beijing Key Lab of Traffic Data Analysis and Mining, Beijing Jiaotong University, Beijing 100044, China; 16112080@bjtu.edu.cn (M.Z.); 16112081@bjtu.edu.cn (Y.G.)

**Keywords:** fine-grained image retrieval, global–local aware feature representation, local descriptors, frequent pattern mining

## Abstract

Fine-grained image retrieval aims at searching relevant images among fine-grained classes given a query. The main difficulty of this task derives from the small interclass distinction and the large intraclass variance of fine-grained images, posing severe challenges to the methods that only resort to global or local features. In this paper, we propose a novel fine-grained image retrieval method, where global–local aware feature representation is learned. Specifically, the global feature is extracted by selecting the most relevant deep descriptors. Meanwhile, we explore the intrinsic relationship of different parts via the frequent pattern mining, thus obtaining the representative local feature. Further, an aggregation feature that learns global–local aware feature representation is designed. Consequently, the discriminative ability among different fine-grained classes is enhanced. We evaluate the proposed method on five popular fine-grained datasets. Extensive experimental results demonstrate that the performance of fine-grained image retrieval is improved with the proposed global–local aware representation.

## 1. Introduction

**Figure 1:**
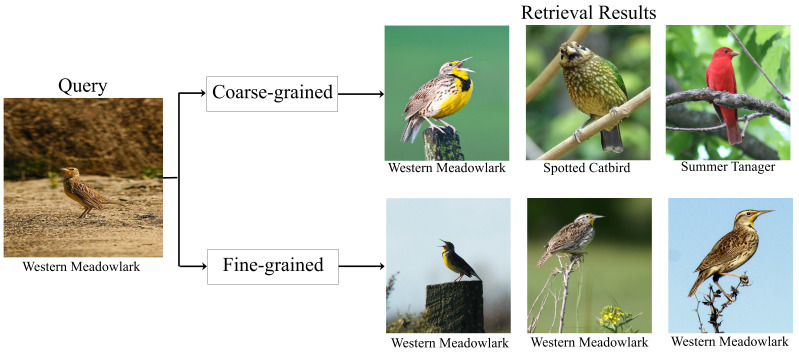
Coarse-grained vs. fine-grained image retrieval. When a user submits a query *“Western Meadowlark”*, coarse-grained image retrieval simply returns results that are related to the category of *“Bird”*, e.g., *“Western Meadowlark”*, *“Summer Tanager”*, or *“Spotted Catbird”*; fine-grained image retrieval returns results that belong to the same subcategory of *“Western Meadowlark”*.

With the rapid advance of the internet and artificial intelligence, image retrieval is one of the challenging research topics, which aims to take one image as a query and retrieve relevant images of the same category [1]. In real-life scenarios, there are two types of retrieval task: coarse-grained and fine-grained image retrieval, as shown in Figure 1. In the former, when a user submits a query *“Western Meadowlark”*, it only returns results that are related to the category of *“Bird”*, e.g., *“Western Meadowlark”*, *“Summer Tanager”*. In the latter, when a user submits a query *“Western Meadowlark”*, the retrieval results must belong to the same subcategory of *“Western Meadowlark”*, even though *“Spotted Catbird”* is similar to *“Western Meadowlark”*. Here, “category” represents “super category”, and “subcategory” refers to “subordinate category”.

Inspired by the breakthrough of deep learning methods [2], coarse-grained image retrieval has achieved great progress in recent years [3]. These methods usually utilize an image encoder (e.g., CNN) to extract global features of images and then devise a metric to measure the similarity of image pairs. Beyond these coarse-grained methods, fine-grained image retrieval faces the challenge of highly similar global geometry and appearance among fine-grained classes; thus, many approaches have been proposed. Xie et al. [4] attempted to construct a hierarchical database relying on handcraft features; subsequently, many supervised methods based on the advanced deep learning models were proposed. Zhang et al. [5] proposed a fine-grained object retrieval scheme that addresses the issues of object localization and centralized ranking loss in a unified framework. Later, they presented a metric learning scheme to address local structure and slow training simultaneously [6]. However, these supervised methods depend on manual annotations that are expensive and labor-intensive. This motivates the development of an unsupervised paradigm, which does not need any training or fine-tuning process. Wei et al. [7] employed a pretrained CNN model [8] to select the relevant deep descriptors. This method is under an unsupervised paradigm that has two merits: on the one hand, it reduces the annotation cost; on the other hand, more importantly, it does not depend on human annotation and, thus, is expected to find more reasonable “labels” automatically. Meanwhile, this method reveals that selecting meaningful deep descriptors is beneficial for removing background or noise. All these approaches achieve promising performance, yet, fine-grained image retrieval is still facing the challenges of learning the discriminative representation and reducing the expensive and labor-intensive annotations.

To address the difficulties, we propose a novel method for fine-grained image retrieval, where global–local aware feature representation is learned. Specifically, we first extract the global feature by selecting the most relevant deep descriptors to localize the saliency object. Meanwhile, in light of the empirical success of frequent pattern mining (FPM) in the field of data mining, we propose to mine the intrinsic correlation of different parts via FPM, and thus extract the representative local feature. The inner correlation of parts is indeed crucial in the fine-grained scenario. For example, *“Western Meadowlark”* and *“Summer Tanager”* belong to the same category but to different subcategories. Common among them is a sharp mouth, and differences are that *“Western Meadowlark”* has a yellow trunk and round tail. In contrast, *“Summer Tanager”* has a yellow–green trunk and square tail. Therefore, the key to distinguishing different subcategories is to mine the correlation. Furthermore, to enhance the discriminative ability among different fine-grained classes, an aggregation feature that learns global–local aware feature representation is designed. Then, we design a similarity score to measure the relevance between the query and image database. Finally, we return the candidate based on the maximum similarity. The main contributions are as follows:

- We learn global–local aware feature representation, which promotes the discriminative property to identify different fine-grained classes.
- We propose to revisit the local feature via FPM, which mines the correlation among different parts.
- We verify our method on five popular fine-grained datasets. Extensive experiments demonstrate the effectiveness of our method.

The rest of our paper is organized as follows: Section 2 discusses the related works about content-based image retrieval, fine-grained image representation, and FPM. Section 3 describes details of the proposed method. Section 4 evaluates the proposed method on five fine-grained datasets. Section 5 concludes this work.

## 2. Related Works

In this section, we briefly review the related works about content-based image retrieval, fine-grained representation, and FPM.

### 2.1. Content-Based Image Retrieval

Given a query, an image retrieval system aims to find similar images from a large-scale dataset. Early image retrieval techniques are not based on visual features but on the textual annotations of images; this means that images are firstly annotated with text and then searched using a text-based method from the traditional database management system [9]. However, its performance is sensitive to the keywords employed by the user and the system. In order to solve the problem, many content-based image retrieval methods have been proposed. These deal with the image content itself, such as color [10], texture [11], and shape [12]. Recently, content-based image retrieval techniques via deep learning have shown great performance [13,14,15]. Xiao et al. [13] proposed a new mechanism based on adversarial examples to stash private images in a deep hash space. Zhang et al. [14] proposed an improved deep hashing framework to enhance the ability of multilabel image retrieval. Cui et al. [15] learned enhanced hash codes for social image retrieval and formulated a unified scalable deep hash learning framework.

Compared with the coarse-grained methods, fine-grained image retrieval aims to retrieve near-duplicate images that belong to the same subcategory. In the literature, many approaches have been proposed and achieved promising results. Xie et al. [4] proposed the challenging topic of fine-grained image search, which is less studied in the multimedia community. Further, they formulated the problem by constructing a hierarchical database and defined an evaluation method. In recent years, deep learning has witnessed the remarkable breakthrough of feature representations. Zhang et al. [5] proposed a fine-grained object retrieval scheme that conquered these issues of object localization and centralized ranking loss in a unified framework. Zhang et al. [6] presented a metric learning scheme to solve local structure and slow training simultaneously. Zeng et al. [16] presented a variant of the cross-entropy loss for enhancing model generalization and promoting retrieval performance. The methods mentioned above are supervised and depend on manual annotations, which are labor-intensive. Wei et al. [7] proposed an unsupervised paradigm that employed a pretrained model to select meaningful deep descriptors as the global feature.

In this work, we focus on the fine-grained image retrieval task. Compared with existing methods, ours learns global–local aware representation, which is unsupervised and promotes the discriminative property to identify different fine-grained classes.

### 2.2. Fine-Grained Feature Representation

Fine-grained feature representation aims at learning the features of hundreds of subcategories. It is a challenging task due to large intraclass variances and small interclass distinctions. Generally, fine-grained feature representation methods can be divided into two groups: two-stage and end-to-end.

The two-stage methods usually utilize handcrafted annotations or localization subnetworks to locate crucial parts and extract the features based on corresponding parts. Early works [17,18] relied on manual annotations to locate semantic parts (e.g., head, mouth) of objects. Zhang et al. [17] learned part-based detectors, and Wei et al. [18] leveraged segmentation methods. However, obtaining such annotations is expensive and labor-intensive, which limits both the scalability and practicality of real-world fine-grained applications. This motivates the development of weakly supervised methods [19,20], which only use image-level annotations. Concretely, Ge et al. [19] built complementary parts models to retrieve information suppressed by dominant object parts. Huang et al. [20] presented an interpretable model and learned a dictionary of object parts.

The end-to-end methods focus on developing deep models to learn more discriminative features. Lin et al. [21] presented the bilinear CNN model, which can efficiently represent an image as a pooled outer product of two CNN features. Another study [22] focused on designing specific loss functions, which drive the whole deep model for learning discriminative fine-grained representations.

In order to promote the discriminative property of fine-grained representation, we propose exploring the inner correlation of different parts. Previous methods only focus on parts, and ignore the inner correlation of different parts.

### 2.3. Frequent Pattern Mining

In data mining, FPM is an important technique, which has received wide attention in many areas such as marketing, advertising, science, and social network analysis. It was firstly introduced by Agrawal et al. [23]. After that, Han et al. [24] designed the frequent pattern tree (FP-tree), which is an extended prefix-tree structure for storing crucial information, and proposed an efficient FP growth algorithm. Benefiting from its development, many researchers applied it to perform computer vision tasks, e.g., image classification [25] and object localization [26].

In this work, we apply FPM to the computer vision task, and mine the intrinsic relationship of different parts. Meanwhile, our method demonstrates that exploring the intrinsic relationship of different parts has a potential for fine-grained feature representation.

## 3. Fine-Grained Image Retrieval via Global and Local Features

In this section, we introduce the proposed method for fine-grained image retrieval. We start with the motivation and overview, and then elaborate details of feature extraction. We also describe the retrieval process. Finally, we discuss the differences with some related works.

### 3.1. Motivation and Overview

Existing fine-grained image retrieval methods focus on capturing the discriminative parts (e.g., ears, tails for birds) for better accuracy. Despite achieving promising results, they show two limitations: (1) Some studies [5,6,16] tend to design specific loss functions under the supervised paradigm, but they are labor-intensive for manual annotation. (2) SCDA method [7] employs the pretrained model to select the meaningful deep descriptors under the unsupervised paradigm but ignores localizing the discriminative local feature. The above challenges have motivated us to design a global–local aware representation. On the one hand, the global feature could coarsely capture the object and discard the background clutters; on the other hand, the local feature could point out the correlation among parts, which are discriminative from other subcategories. Besides, we tackle the issue under an unsupervised paradigm, which is more realistic and proper for the fine-grained scenario.

**Figure 2:**
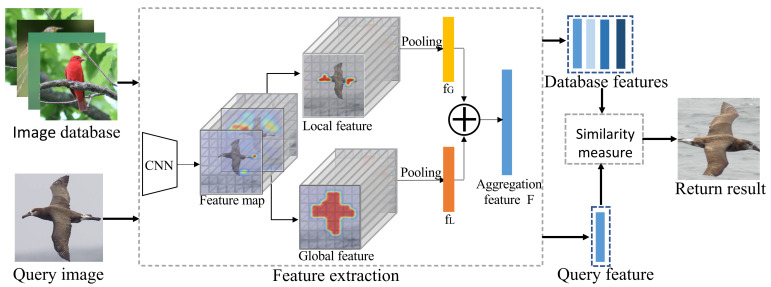
The schema of the proposed method. For each image in a database, we learn its global–local aware representation. Similarly, when given a query image, we also learn its global–local aware representation. Then, we measure the relevance between the query and database images by a similarity score. Finally, we return the candidate based on the score.

As shown in Figure 2, for each image in a database, we learn its global–local aware representation. Concretely, the global feature is extracted by localizing the saliency object, and the local feature is extracted via mining the intrinsic correlation of parts. Then, the two level features are aggregated together to enhance the discriminative ability for the subtle differences among fine-grained classes. Similarly, when given a query image, we also learn its global–local aware representation. Then, we design a similarity score to measure the relevance between the query and database images. Finally, we return the candidate based on the similarity score.

### 3.2. Global Feature Learning

Global feature extraction firstly localizes the object via selecting the relevant deep descriptors, and then extracts its feature. The details are as follows. Feed an image into CNN; the activation of a convolution layer is denoted as a tensor *T* of size h×w×d, where *h*, *w*, *d* represent the height, width, and depth, respectively. *T* includes *d* feature maps of size h×w. From another point of view, *T* can be also considered as having h×w cells, and each cell is a *d*-dimension deep descriptor. For example, we input an image of size 224×224 into the pretrained VggNet-16 [8]; in the layer of $pool_{5}$, we obtain a 7×7×512 activation tensor; on the other hand, 49 deep descriptors of 512-dimension are obtained. When the activation tensor is produced, the following task is how to obtain the object.

Considering that a position is expected to belong to an object if multiple channels fire there, we add up the activated tensor through the *d* direction. In this way, a 3-D h×w×d tensor becomes a 2-D h×w tensor, called “aggregation map”, *A*. For *A*, there are h×w summed responses, corresponding to h×w positions. Based on the observation [7], the higher response a position has, the more possibility it belongs to an object. Thus, we firstly calculate the mean value of *A* as the threshold, δ. Then, we scan *A* to generate the mask map $A^{\prime }$:

(1) $$\begin{array}{c} A_{i,j}^{\prime }=\left\{\begin{array}{c} 1,\;\mathrm{if}\;A_{i,j}>\delta \\ 0,\;\mathrm{otherwise}, \end{array}\right. \end{array}$$

where (i,j) is the position index of feature map, 1≤i≤h, and 1≤j≤w.

After $A^{\prime }$ is generated, we utilize the flood fill algorithm [7] to retain the largest connected component, which represents the salient object. Then, we employ the pooling operation to learn the global feature, $f_{G}$.

### 3.3. Local Feature Learning

**Figure 3:**
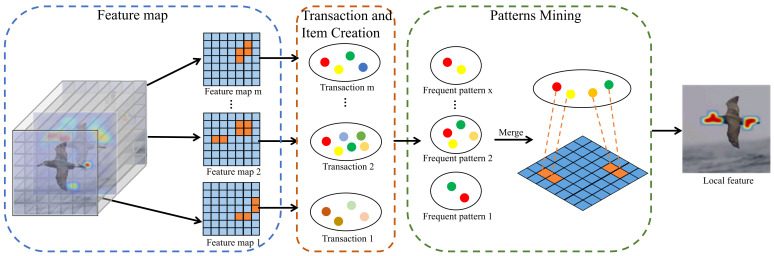
The procedure of local feature learning. We input an image into the CNN and obtain feature maps. By threshold operation, we retain activated positions (orange dots) and discard inactivated positions (blue dots). Then, we convert feature maps and activated positions into transactions and items, and mine the frequent patterns. Finally, the local feature is extracted.

Previous local feature learning works [19,20] focus on localizing the discriminative parts but ignore the correlation among parts. In this work, we propose to mine the inner relationship of different parts via FPM, which is described in Figure 3. To make this paper self-contained, we first restate the preliminary of FPM; then, we depict how the feature maps and positions are converted into transactions and items and how the meaningful patterns are discovered.

#### 3.3.1. Preliminary

In data mining, FPM is a prevalent algorithm that aims to reveal the essential characteristics of things by analyzing a large number of data and extracting the correlation. FPM originated from market analysis. The supermarket manager analyzes the past transaction records and explores customers’ shopping motivation and habits to maximize the profit and formulate a targeted marketing strategy, e.g., ”bread → milk (80%)” means that 80% of customers buy milk while buying bread. This discovery can provide vital support to make business decisions, such as promotions and mall layout.

Formally, let $I={\left\{a_{i}\right\}}_{i=1}^{n}$ be a set of items, where $a_{i}$ denotes the *i*-th item. Let $T={\left\{t_{i}\right\}}_{i=1}^{m}$ be a set of transactions, where $t_{i}$ represents the *i*-th transaction, and $t_{i}$ is also a subset of *I*. Given an itemset P⊆I, we define the support value of *P* as

(2) $$supp\left(P\right)=\frac{k}{m}=\frac{\left|\{t_{i}|t_{i}\in T,P\subseteq t_{i}\}\right|}{m}\in \left[0,1\right],$$

where |·| measures the cardinality. Considering that, in our task, *m* is a constant and denotes the number of feature maps, we directly set it as

(3) supp(P)=k,

where *P* is a frequent pattern when supp(P)≥minsupp. Here, minsupp refers to a threshold.

FPM adopts the divide-and-conquer strategy, including two steps: frequent pattern tree construction (FP-tree construct) and frequent pattern growth (FP growth).

**FP-tree construct:** we first generate frequent items through minsupp and compress them in an FP-tree. Each node of the FP tree consists of two parts: label and count. The construction process is as follows:

- Create the root of FP-tree.
- Scan the transactions. For each transaction, sort the reserved items in order of decreasing support.
- Create a branch for the first transaction and insert subsequent transactions into the FP-tree one by one. Specifically, when inserting a new transaction, check whether a shared prefix exists between the transaction and the existing branches. If so, increase the count of all shared prefix nodes by 1 and create a new branch for the items after the shared prefix.

**FP growth:** when the FP-tree is constructed, we aim to mine the frequent patterns from the FP-tree. Details are as follows:

- For any frequent item, construct its conditional pattern base. Here, the conditional pattern base refers to the FP-subtree corresponding to the node we want to mine as the leaf node. To obtain the FP-subtree, we set the count of each node in the subtree to equal the count of the leaf node, and delete the nodes whose count is lower than minsupp.
- Mine frequent patterns recursively after obtaining the conditional pattern base. When the conditional pattern base is empty, its leaf node is the frequent pattern; otherwise, enumerate all combinations and connect them with the leaf node to obtain frequent patterns.

#### 3.3.2. Transaction and Item Creation

The most critical step is converting the computer vision task into a data mining task. In our scenario, each feature map is converted into a transaction, and each activated position corresponds to an item. For example, if there are six activated position in the *j*-th feature map, from the perspective of data mining, the corresponding transaction $t_{j}$ would contain six items, i.e., $t_{j}=\left\{a_{1},a_{2},a_{3},a_{4},a_{5},a_{6}\right\}$. In practice, we input an image into the pretrained model, and the image is mapped into a set of feature maps via the convolution operation. For each feature map, we calculate its mean value as the threshold. Then, we scan the pixels of the feature map, and if the response of a pixel is higher than the mean value, the pixel will be activated. In this manner, we retain activated positions and discard inactivated positions. Finally, each feature map and activated pixel are regarded as a transaction and item, respectively.

#### 3.3.3. Frequent Pattern Mining

Formally, let $I^{\prime }={\left\{a_{i}^{\prime }\right\}}_{i=1}^{n}$ be a set of activated positions, where $a_{i}^{\prime }$ denotes the *i*-th activated position. Let $T^{\prime }={\left\{t_{i}^{\prime }\right\}}_{i=1}^{m}$ be a set of feature maps, where $t_{i}^{\prime }$ represents the *i*-th feature map, and is also a subset of $I^{\prime }$. Given an itemset $P^{\prime }\subseteq I^{\prime }$, $P^{\prime }$ is considered frequent if $supp(P^{\prime })\geq minsupp$. A proper choice of minsupp depends on the distribution of the handled dataset. Thus, we leave minsupp as a hyperparameter and will empirically discuss it in Section 4.1.

The overall process of local feature learning is shown in Figure 3. Firstly, we input an image into the CNN and obtain feature maps. By threshold operation, we retain activated positions and discard inactivated positions. Note that the activated positions and the inactivated positions are represented by orange and blue dots, respectively. Then, we transform feature maps and activated positions into transactions and items, and mine the frequent patterns via FPM. Finally, we employ a pooling operation to extract the local feature $f_{L}$ from the patterns. The complete process is summarized in Algorithm 1.
**Algorithm 1** Revisiting Local Feature Learning via FPM**Input:** Feature maps $\left(T^{\prime }\right)$, Activated positions $\left(I^{\prime }\right)$. **Output:** Local feature $(f_{L}$).  1: **FP-tree construct:** generate frequent positions through minsupp, and compress them in an FP-tree;  2: **FP growth:** for any frequent position of FP-tree, construct its conditional pattern base and mine frequent patterns recursively;  3: By the pooling operation, extract the local feature $\left(f_{L}\right)$ from the frequent patterns.

### 3.4. Retrieval Procedure

In order to tackle the retrieval task, when given a query, we aim at searching the top *K* nearest neighbors from the image database. Precisely, for a query, we extract its global feature $f_{G}$ and local feature $f_{L}$. Likewise, for each image in the database, we extract its global feature $f_{G}$ and local feature $f_{L}$. Then, we calculate the aggregated feature F by

(4) $$\begin{array}{c} F=f_{G}+\alpha f_{L}, \end{array}$$

where α is a weight balancing the effect of different features, and more details are found in Section 4.1.

To measure the similarity between the query and database image, we define the similarity score as

(5) $$S\left(F_{Q},F_{D}\right)=\frac{{F_{Q}}^{T}F_{D}}{|F_{Q}\left|\right|F_{D}|},$$

where $F_{Q}$ and $F_{D}$ denote the aggregated feature of query and the aggregated feature of database image, respectively. Finally, we return the candidate based on the score.

### 3.5. Discussion

While our method performs feature extraction via a global–local aware feature representation and is devised for fine-grained image retrieval, it bears certain correlations with some related works. We highlight them in the following.

**Difference with existing unsupervised method.** In contrast with [7], though we share the similar idea of selecting the related deep descriptors to learn the global feature, we apply the idea of learning local features to point out the intrinsic relationship of different parts, which enhances the discrimination among subcategories and is complementary with the global feature.

**Difference with existing supervised methods.** Compared with existing supervised approaches [5,6] driving the deep models by designing loss functions, we perform the fine-grained image retrieval in a purely unsupervised setting, which is more realistic for the fine-grained scenario. Meanwhile, the proposed method demonstrates that incorporating frequent pattern mining strategy with the pretrained model has potential to learn the fine-grained representation.

## 4. Experiments

In this section, we evaluate the performance of our proposed method. We first introduce the data, evaluation metrics, implementation details, and compared methods. Then, we describe the comparison of our method with some state-of-the-art methods. In order to better understand the influence of each component, we conducted an ablation study. Finally, we visualize some qualitative results.

### 4.1. Experimental Settings

**Datasets.** We evaluate our model on five fine-grained datasets. In the following, we briefly introduce these datasets; Table 1 shows the statistics.

- **Dataset 1: CUB200-2011** [27] contains 11,788 images of 200 subcategories.
- **Dataset 2: Stanford Dog** [28] contains 20,580 images of 120 subcategories.
- **Dataset 3: Oxford Flower** [29] contains 8189 images of 102 subcategories.
- **Dataset 4: Aircraft** [30] contains 10,200 images of 100 subcategories.
- **Dataset 5: Car** [31] contains 16,185 images of 196 subcategories.

**Evaluation Metrics.** We used the commonly used evaluation metric, mean average precision (mAP) score, as the metric, to evaluate the performance [32]. Concretely, we first calculated the average precision (AP) score for each query, and then calculated their mean value as the mAP score. Formally, mAP is computed by

(6) $$\begin{array}{cc} \mathrm{mAP}= & \frac{1}{Q}\sum_{q=1}^{Q}\mathrm{AP}(q), \end{array}$$

(7) $$\begin{array}{cc} \mathrm{AP}= & \frac{1}{R}\sum_{k=1}^{n}\left(p\left(k\right)\cdot \mathrm{rel}(k)\right), \end{array}$$

where *Q* represents the number of images to be retrieved, *R* denotes the total number of relevant images, *k* refers to the sorting position, p(k) is the accuracy of the first *k* results, and rel(k) indicates whether the images at position *k* are related.

**Figure 4:**
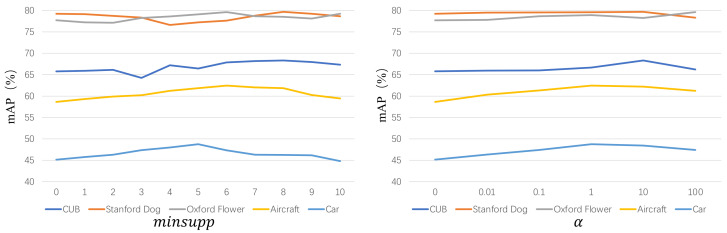
Hyperparameter analysis. The above figures show the mAP values with different parameters minsupp (for FPM) and α (for balancing the effect of global and local features).

**Implementation Details.** All experiments are conducted on a 64-bit Ubuntu 16.04 with 2 Intel 2.40 GHz CPUs, 256 GB memory, and 6 NVIDIA Tesla GPUs. For a fair comparison, we employ the publicly available pretrained VggNet-16, which is initialized with the weights on ImageNet [33]. Note that VggNet-16 can be replaced with any CNN model. For each dataset, the hyperparameters minsupp and α are searched in the scopes {0, 1, 2, ⋯, 10} and {0, 0.01, 0.1, ⋯, 100}, respectively. Figure 4 shows the details. In practice, we input the image of size 224×224 into the VggNet-16, and obtain its salient object by the mask operation. Then, we concatenate the max-pooling (512-dimension) and average-pooling (512-dimension) of the saliency as the global feature, $f_{G}$ (1024-dimension). For local feature learning, we first mine the frequent patterns by the frequent pattern mining algorithm. Then, we concatenate the max-pooling (512-dimension) and average-pooling (512-dimension) of patterns as the local feature, $f_{L}$ (1024-dimension). Finally, we aggregate the global and local features to obtain the global–local aware feature.

**Methods for Comparison.** We divide the baseline approaches into two categories, including six coarse-grained and four fine-grained methods. Details are as follows:

- **SIFT_FV** (coarse-grained): The SIFT features are conducted with Fisher Vector encoding as the handcrafted feature-based retrieval baseline. The parameters of SIFT and FV used in our experiment follow [4]. The feature dimension is 32,768. In addition, we replace the whole image with the region within the ground truth bounding box as the input, which is named “**SIFT_FV_gtBBox**”.
- **Fc_8** (coarse-grained): For the Fc_8 baseline, because it requires the input images at a fixed size, the original images are resized to 224×224 and then fed into VggNet-16. Similarly, we replace the whole image with the region within the ground truth bounding box as the input, which is named “**Fc_8_gtBBox**”.
- **Pool_5** (coarse-grained): For the Pool_5 baseline, it is extracted directly without any selection procedure. We concatenate the max-pooling (512-dimensional) and average-pooling (512-dimensional) into avg+maxPool (1024-dimensional), as the image feature. In addition, VLAD and FV are employed to encode the selected deep descriptors, and we denote the two methods as **SelectVLAD** and **SelectFV**, which have larger dimensionality.
- **SPoC** [34] (coarse-grained): SPoC aggregates local deep features to produce compact global descriptors for image retrieval.
- **CroW** [35] (coarse-grained): CroW presents a generalized framework that includes cross-dimensional pooling and weighting steps; then, it proposes specific nonparametric schemes for both spatial and channelwise weighting.
- **R-MAC** [36] (coarse-grained): R-MAC builds compact feature vectors that encode several image regions without feeding multiple inputs to the network. Furthermore, it extends integral images to handle max-pooling on convolutional layer activations.
- **SCDA** [7] (fine-grained): SCDA utilizes the pretrained CNN model to localize the main object, and meanwhile discards the noisy background. Then, SCDA aggregates the relevant features and reduces the dimensionality into a short feature vector.
- **CRL** [5] (fine-grained): CRL proposes an efficient centralized ranking loss and a weakly supervised attractive feature extraction, which segments object contours with top-down saliency.
- **DCLNS** [6] (fine-grained): DCLNS presents a metric learning scheme, which contains two crucial components, i.e., Normalize-Scale Layer and Decorrelated Global-aware Centralized Ranking Loss. The former eliminates the gap between training and testing as well as inner-product and the Euclidean distance, while the latter encourages learning the embedding function to directly optimize interclass compactness and intraclass separability.
- **PCE** [16] (fine-grained): PCE proposes a variant of cross entropy loss to enhance model generalization and promote retrieval performance.

### 4.2. Comparisons with State-of-the-Art Methods

Table 2 compares the proposed method with established methods, including coarse-grained and fine-grained image retrieval methods.

**Comparison with Coarse-grained Methods.** The proposed method outperforms the coarse-grained methods on *CUB200-2011*, *Stanford Dog*, *Oxford Flower*, and *Aircraft* datasets. However, on the *Car* benchmark, the performance of coarse-grained methods is better than the proposed method. The reason is that fine-grained methods pay attention to subtle features, but coarse-grained methods focus on expressing the whole image; for the *Car* dataset, since the cars belong to rigid bodies, the variations in the same subcategory are subtle. Besides, the distinctions among different subcategories are large enough. So, coarse-grained methods achieve better performance.

**Comparison with Fine-grained Methods.** The proposed method performs better than the existing fine-grained methods in almost all experiments. The reason is that the proposed method explores the inner correlation of different local parts. Meanwhile, it jointly considers global–local two-level feature representations. Such two-level feature representations ensure the compactness characteristic of the same subcategory and discriminative property of different subcategories. Previous works have been devoted to solving this task via designing specific loss functions to learn the local feature [5,6,16] or selecting the relevant deep descriptors to obtain the global feature [7], but they all ignore the complementation of global and local features.

### 4.3. Ablation Study

In order to better understand the different components of the proposed method, we conducted the ablation study on five datasets. The results are shown in Table 3. Note that “Original”, “Global-stream”, “Local-stream”, “Global–Local aware” refer to performances of the original image, global representation learning, local representation learning, and proposed approach, respectively. From Table 3, we can observe the following:

- Compared with the “Original”, “Global-stream”, and “Local-stream”, the proposed method boosts performance significantly. This is mainly because global–local aware feature representation combines “Global-stream” and “Local-stream” simultaneously. On the one hand, global feature learning could localize the saliency areas and discard the noises correctly. On the other hand, local feature learning could learn the coactivated local features, which are mutually complementary with the “Global-stream”, to enhance the discriminative property.
- Compared with “Global-stream”, the retrieval results of “Local stream” are also promising. The reason is that subtle visual differences exist in different subcategories. “Global-stream” encodes the basic-level object features but ignores the subtle features, while “Local stream” captures the subtle and minute differences among different subcategories, and mines the inner relationship.

**Figure 5:**
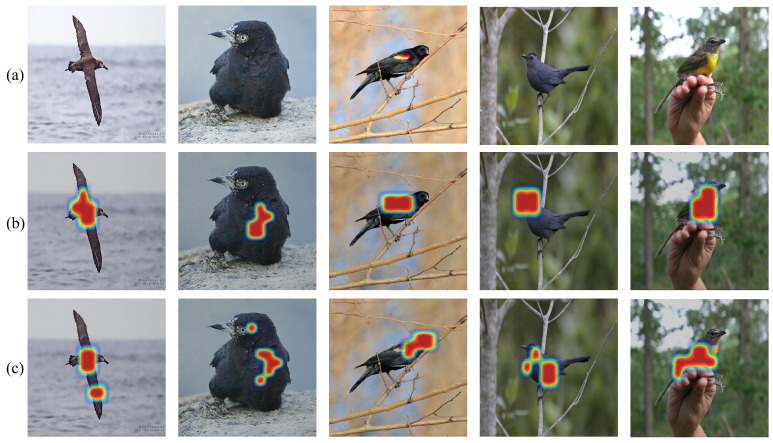
Heat map comparison in **CUB200-2011** dataset. From top to bottom: (**a**) original image, (**b**) heat map of the global feature, (**c**) heat map of the local feature.

Furthermore, to better understand global–local aware feature representation, we visualize the two-level features in Figure 5. From top to bottom: the original image, the heat map of global feature, the heat map of local feature. This shows that the global feature focuses on the main salient object and discards noise, while the local feature captures multiple interpretable semantic local details, e.g., belly, tail, and feet of the bird. In addition, these local details are coactivated and could be assembled to provide a discriminative fine-grained feature.

## 5. Conclusions and Future Work

In this paper, we propose a novel fine-grained image retrieval method, where global–local aware feature representation is learned. Specifically, the proposed method performs global feature learning by localizing the object, and extracts the local feature via mining the intrinsic relationship. We evaluate the proposed method on five popular fine-grained datasets. Extensive experimental results demonstrate the effectiveness.

In future research, there are some topics that we will investigate. (1) **Fine-grained hashing**. As hundreds of thousands of images are uploaded to the Internet every day, more and more large-scale and well-constructed fine-grained datasets have been released, e.g., [37,38,39]. Therefore it is necessary to adopt an efficient and compact code to manage the large-scale data. Hashing is an efficient tool for large-scale data retrieval. Compared with traditional searching methods, hashing has lower time and space complexity. Consequently, fine-grained hashing is a promising direction and deserves further exploration. (2) **Fine-grained cross modal retrieval**. In recent years, different modalities of media, such as image, text, and video, have grown rapidly on the Internet. It is prevalent to enable computers to understand, match, and transform such cross-modal data; thus, cross-modal retrieval is one of the most fundamental topics. Meanwhile, in many real-world scenarios—e.g., automatic biodiversity monitoring, climate change evaluation, intelligent retail, intelligent transportation—the retrieval results are required to be further fine-grained. Thus, fine-grained cross modal retrieval is promising and deserves further research efforts in the future.

## Figures and Tables

**Table 1:** Summary of datasets. Note that “BBox” denotes whether this dataset provides object bounding boxes. “Part Anno” indicates providing key part localizations.

Dataset	Images	Categories	BBox	Part Anno
CUB200-2011	11,788	200	✓	✓
Stanford Dog	20,580	120	✓	
Oxford Flower	8189	102		
Aircraft	10,200	100	✓	
Car	16,185	196	✓	

**Table 2:** mAP accuracy (%) comparison with coarse-grained (the upper part) and fine-grained methods (the lower part) on five datasets. The best results are highlighted by bold face.

Dataset	CUB	Stanford Dog	Oxford Flower	Aircraft	Car
SIFT_FV	8.07	16.38	36.19	37.44	24.11
SIFT_FV_gtBBox	14.29	21.15	-	46.87	40.34
Fc_8	48.10	72.69	60.37	35.00	25.77
Fc_8_gtBBox	55.34	76.61	-	41.25	37.45
Pool_5	63.66	75.55	74.05	53.61	41.86
SelectFV	59.19	73.74	73.60	54.68	41.60
SelectVLAD	62.51	74.43	76.86	56.37	43.84
SPoC	47.30	55.69	70.05	48.95	33.88
CroW	59.69	68.33	76.16	58.62	51.18
R-MAC	59.02	66.28	78.19	54.94	**52.98**
SCDA	65.79	79.24	77.70	58.64	45.16
CRL	67.23	76.43	78.65	60.21	48.16
DGCRL	67.97	78.25	79.21	59.34	49.67
PCE	66.79	78.43	78.23	60.64	47.16
**Ours**	**68.32**	**79.68**	**79.62**	**62.45**	48.76

**Table 3:** Ablation study on five datasets. “Original”, “Global-stream”, “Local-stream”, “Global–Local aware” refer to performances of the original image, global representation learning, local representation learning, and proposed approach, respectively.

Dataset	CUB	Stanford Dog	Oxford Flower	Aircraft	Car
**Original**	56.21	64.63	58.31	50.69	40.46
**Global-stream**	65.79	79.24	77.70	58.64	45.16
**Local-stream**	67.78	77.23	78.98	60.98	48.35
**Global–Local aware**	**68.32**	**79.68**	**79.62**	**62.45**	**48.76**

## Data Availability

Not applicable.
